# Enhancing egg production and quality by the supplementation of probiotic strains (*Clostridium* and *Brevibacillus*) *via* improved amino acid digestibility, intestinal health, immune response, and antioxidant activity

**DOI:** 10.3389/fmicb.2022.987241

**Published:** 2022-09-13

**Authors:** Uchechukwu Edna Obianwuna, Kai Qiu, Xin-yu Chang, Hai-jun Zhang, Jing Wang, Guang-hai Qi, Tie-hu Sun, Yong-bo Su, Shu-geng Wu

**Affiliations:** ^1^National Engineering Research Center of Biological Feed, Institute of Feed Research, Chinese Academy of Agricultural Sciences, Beijing, China; ^2^China Oil Foodstuffs Corporation (COFCO) Nutrition and Health Research Institute, Beijing, China; ^3^Technology Department, China Oil Foodstuffs Corporation (COFCO) (Beijing) Feed Technology Company Limited, Beijing, China

**Keywords:** probiotics, laying hens, egg quality, gut health, immune response, antioxidant capacity

## Abstract

This study focused on evaluating the influence of *Clostridium butyricum* and *Brevibacillus* strains on egg production, egg quality, immune response and antioxidant function, apparent fecal amino acid digestibility, and jejunal morphology when supplemented as probiotics in the diets of laying hens in the peak phase. A total of 288 healthy 30-week-old Hy-Line Brown laying hens were arbitrarily assigned to four dietary groups, which included control diet and control diet supplemented with 0.02% *C. butyricum* zlc-17, *C. butyricum* lwc-13, or *Brevibacillus* zlb-z1, for 84 days. The results showed that dietary *C. butyricum* and *Brevibacillus* sp. exerted a positively significant influence (*P* ≤ 0.05) compared to the control group on the performance, egg quality, and physiological response of the birds. The diets could reduce mortality rate and enhance (*P* ≤ 0.05) egg weight and egg mass, egg production rate, and feed efficiency. Further analysis suggested that the probiotic strains can enhance (*P* ≤ 0.05) eggshell quality, Haugh unit, thick albumen content, and albumen height. Also, probiotics enhanced (*P* ≤ 0.05) the antioxidant status *via* increased antioxidant enzymes and jejunal morphology as evidenced by increased villi surface area (VSA), the ratio of villi height to crypt depth, villi width, and villi height, and a significant reduction in crypt depth. Besides, nutrient absorption and retention were enhanced, as apparent fecal amino acid digestibility of key essential amino acids was substantially improved in the diet-based group. The concentrations of immunoglobulin M and A (IgM and IgA) increased significantly (*P* ≤ 0.05) in the probiotics group and the same effect was notable for complement proteins (C3) and immune organ (Spleen). Conclusively, the supplementation of *Clostridium butyricum* zlc-17 in comparison to *Clostridium butyricum* lwc-13 and *Brevibacillus* zlb-z1 strains significantly (*P* ≤ 0.05) promoted the antioxidant status, modulated the intestinal structure, enhanced amino acid digestibility, and regulated the immunity index of the laying hens, which finally improves the laying performance and egg quality of the laying hens.

## Introduction

Feed additives are often employed in poultry nutrition to enhance the health status of the birds, growth performance, and efficiency of production (Markowiak and Śliżewska, 2018). The utilization of synthetic antibiotics as feed additives in animal production is targeted mainly toward gut health; this appears beneficial, but such may not be the case in laying hen’s production due to issues related to egg safety. Also, the abrogation of antibiotic uses in animal diets according to European Parliament and Council Regulation EC No. 1831/2003 due to its adverse effects, such as drug resistance, residue effect, and environmental pollution (Wang et al., 2015), lends more evidence to its non-use in animal production despite its beneficial effect on animal health. To this end, other countries including China, the United States, and South Korea have also adopted antibiotic-free diets in animal production. In order to maintain an equilibrium between egg safety for consumers and animal health, feed additives, including probiotics, prebiotics, synbiotics, and organic acid, which tend to stimulate favorable growth and immune function in farm animals without adverse effects on animal product quality, have been advocated for Al-Khalaifah (2018).

Probiotics are often considered as “Live” micro-organisms, which when supplied in a substantial amount, provide the host with an improved health and welfare status (Food and Agriculture Organization and World Health Organization Expert Consultation, 2001). The underlying mechanism of probiotic actions, including the production of metabolites (short-chain organic fatty acids), immunostimulatory effects, alteration of gastrointestinal flora, and exclusive competitive binding to receptors (Sherman et al., 2009; Ahasan et al., 2015), accounts for the myriad of its positive influence on animal production and health in the poultry industry. Probiotics have been found to enhance laying performance (Mikulski et al., 2020; Macit et al., 2021; Xu et al., 2022), egg quality (Deng et al., 2021; Wang J. et al., 2021; Ray et al., 2022), immune response (Song et al., 2019; Deng et al., 2021; Pan et al., 2022), gut health (Abdel-Latif et al., 2018; Yang et al., 2020), and reduced oxidative stress response (Deng et al., 2021; Xu et al., 2022). Nevertheless, some studies reported that probiotics had no influence on egg production (Arpášová et al., 2016; Shi et al., 2020), egg quality: albumen quality (Souza et al., 2021) and eggshell quality (Wang W. W. et al., 2020), and antioxidant capacity (Forte et al., 2016). The probiotic strain used in the diet may be a contributory factor to the non-significant effect. Studies demonstrated that probiotics could be supplemented in the diet of laying hens as a single strain or a combination of different strains (Xiang et al., 2019; Yang et al., 2020). In the poultry industry, microorganisms often used as probiotics include colonizing species of *Enterococcus. Streptococcus, Bacillus, Lactobacillus*, and *Clostridium*.

*Clostridium butyricum* (CB) spores are highly stable anaerobic endospore-forming gram-positive bacteria, with a capacity to withstand higher temperatures and bile concentration (Kong et al., 2011); thus, they might be utilized in the diet of laying hens as safe feed additives. Previous pieces of literature showed that supplementation of CB in the diet of laying hens at different levels, 0.5 g/kg (Xiang et al., 2019), 0.9 g/kg (Wang et al., 2020), 1 × 10^9^ CFU/kg (Wang et al., 2021), and 5 × 10^8^ CFU/g (Zhan et al., 2019), exerted no negative effect, which lends more evidence of CB as safe feed additives. Our preliminary studies also revealed that CB could be supplemented at 0.02%. In previous research, the positive influence of CB on egg production and egg quality (Xiang et al., 2019; Zhan et al., 2019; Wang Y. et al., 2021) has been reported. Furthermore, *C. butyricum* can positively influence intestinal morphology and health (Zhang et al., 2011; Xiang et al., 2019; Wang Y. et al., 2021), probably because it can act as a source of nutrients for intestinal epithelium and modulate intestinal microflora and intestinal pH (Meimandipour et al., 2010; Zhang et al., 2016; Takahashi et al., 2018). Also, *C. butyricum* possesses the potential to improve antioxidant capacity and immune function (Zhan et al., 2019; Wang Y. et al., 2021) and nutrient absorption and utilization *via* the stimulation of enzymes and nutrient transporters (Wang W. W. et al., 2020). Therefore, the potential of CB to modulate gut health, antioxidant, and immune function may account for its beneficial influence on laying performance and egg quality. However, the *C. butyricum* species used in this study may differ from previous ones, where probably these species were reconstituted and designed to be more suitable for the peak phase of laying hens. In the same line, *Bacillus* strains are stable in an acidic gut environment, form biofilm in the small intestine, could be delivered in the form of spores (Jeong and Kim, 2014), and thus could be used as feed additives. The *Bacillus* spores were previously adopted as a feed additive to increase egg production (Mazanko et al., 2018; Zhou et al., 2020) and albumen quality (Wei et al., 2020; Zhou et al., 2020; Darsi and Zhaghari, 2021). The potential of *Bacillus* sp. to enhance nutrient utilization (Souza et al., 2021), intestine morphology (Yang et al., 2020; Wang J. et al., 2021), and serum antioxidant capacity (Zhou et al., 2020) has also been demonstrated. Thus, *Bacillus* strains can be supplemented in the diets of laying hens because of the potential to maintain the physiological status of the animals, which could translate to improved laying performance and egg quality. Furthermore, strains of *Brevibacillus* are producers of antibacterial and antifungal agents (Panda et al., 2014) and, hence, can be used for biological control. Previous reports showed that *Brevibacillus brevis* (FJAT-1501-BPA) can suppress the abundance of *Staphylococcus aureus, E. Coli* K88, and *Salmonella typhimurium* (Ge et al., 2009) while *Brevibacillus laterosporus texasporus* enhanced the intestine health of broiler birds (Purba et al., 2020), suggesting that the strain has the potential to be used as a probiotic in animal feeding. However, there exists a dearth of information on the utilization of *Brevibacillus* sp. in the diet of laying hens.

There are several studies on *Clostridium* sp. in laying hens, but the strains used in this study are newly created and designed specifically for peak-laying hens; moreover, studies on the effects of *Brevibacillus* sp. on egg production rate, egg quality, physiological status, and intestinal morphometric of laying hens rarely exist. Therefore, the current study investigated the dietary influence of *Clostridium butyricum* and *Brevibacillus spores* on egg quality, laying performance, amino acid digestibility, immune response, intestinal morphology, and antioxidant function in Hy-Line Brown laying hens.

## Materials and methods

### Ethics statement

The Animal Ethics and Use Committee of the Feed Research Institute of the Chinese Academy of Agricultural Sciences, Beijing, China consented to all protocols utilized in the current study with the animal ethics approval number CAAS. No.: 20200507S0600103.

### Experimental design

Hy-Line Brown laying hens (*n* = 288, 30-week old) at the peak-laying phase (initial egg production rate = 89.0 ± 1.5%) with similar laying rates were arbitrarily assigned to one of four dietary groups, each of which consists of six replicates (*n* = 72 laying hens). The feeding trial included a 12-week test phase and a 2-week acclimation or a feed transfer period (lasted for 14 weeks; 30–44 weeks of age). On a daily basis, the laying hens were offered fresh water and feed *ad lib*, the birds were given routine vaccination, and management was based on the Hy-line International Online Management Guide. Throughout the feeding trial period, the laying hens were managed under a controlled house environment (on a daily basis): humidity (50–80%) and 16 h of light and temperature (24^°^C). During the feeding trial, the laying hens had stable good health as there was no disease outbreak, and invariably, no medications were offered.

The diet groups consist of the control diet without supplementation of probiotics, basal diet + 0.02% of *C. butyricum* (zlc-17), basal diet + 0.02% of *C. butyricum* (lwc-17), and basal diet + 0.02% of *Brevibacillus* (zlb-z1). The viable count of three kinds of probiotic products is the same, i.e., 1 × 10^9^ CFU/g. Prior to the feeding trial, the birds were fed laying hens’ diets; a basal diet of mashed corn and soybean. This diet was sufficient in all nutrients and met the necessary standards. The information regarding basal diet, nutrient level, and nutrient composition, is presented in Table 1, and the diet was in accordance with the nutrient formulation guide of the National Research Council (National Research and Council, 1994). The probiotics were purchased from COFCO Nutrition and Health Research Institute, Beijing, China.

**TABLE 1:** The Composition and nutrient levels of the basal diet.

Ingredients	Content(%)
Corn	63.40
Soybean meal	25.46
Stone powder	8.76
DL-methionine	0.18
Dicalcium phosphate	1.60
Salt	0.16
Premix (choline chloride)	0.25
Sodium sulfate	0.17
Phytase	0.02
Total	100.00
Nutrient content	**%**
Crude protein	16.50
Calcium	3.50
Total phosphorus	0.60
Available phosphorus	0.39
Metabolizable energy, MJ/kg	11.23
SID methionine	0.434
SID lysine	0.796
SID tryptophan	0.176
SID threonine	0.560
SID methionine + cysteine	0.653
SID isoleucine	0.666
SID cysteine	0.240
SID valine	0.746
SID arginine	1.030
SID leucine	1.414
SID serine	0.776
SID glycine	0.616

The values are calculated values. AME, apparent metabolizable energy. SID, Standard ileal digestibility; Met + Cys, Methionine + Cysteine. 2 Vitamin and mineral premix provided the following per kg of diets: VA: 12,500 IU; VD3: 4,125 IU; VE: 15 IU; VK: 2 mg; VB1: 1 mg; VB2: 8.5 mg; VB6: 8 mg; VB12: 5 mg; calcium pantothenate: 50 mg; niacin: 32.5 mg; biotin: 2 mg; folic acid: 5 mg; choline: 500 mg; Mn: 65 mg; I: 1 mg; Fe: 60 mg; Cu: 8 mg; Zn: 66 mg.

### Performance measurement

During the feeding experiment, which lasted for 12 weeks, the following records were taken per replicate on a daily basis, egg number, egg weight, damaged eggs, and mortality rate, and data collection on feed intake was done on a fortnight basis. Based on the collected data, the following calculations were deduced; hen day production (HDP), feed conversion ratio (FCR), average feed intake (ADFI), average egg weight (AEG), and egg mass for the whole trial period (π × VW × VH).

### Sample collection and laboratory analysis of blood

After the feeding trial (12 weeks), 24 birds (six from each group, one per replicate) were separated and kept in other cages and subjected to a 12-h fast prior to slaughter. About 5 mL of blood was drawn from the wing vein for the measurement of whole blood and serum indices. The blood samples collected in a micro-anticoagulant tube were kept slant at a fixed point for a period of 30 min and then centrifuged (300 × g for 15 min) (Tang et al., 2018). The obtained serum was transferred to Eppendorf tubes (1.5 mL) and kept at a low temperature (−20^°^C). The blood samples were transported to the laboratory in an ice pack within 1 h of collection for hematology analysis.

An automated hematological analyzer (Model: BC-2800 Vet, Mindray, Shenzhen, China) was used for the hematological analysis. Prior to serum indices analysis, the serum was thawed and maintained at a low temperature (4^°^C) to prevent enzyme activation. The concentrations of malondialdehyde (MDA), glutathione transferase (GST), catalase (CAT), total antioxidant capacity (T-AOC), total superoxidase dismutase (T-SOD), and glutathione peroxidase (GSH-Px) in the serum were analyzed with the corresponding ELISA kits (A003-1, ml023160, A007-1-1, ml063644, A001-1-1, and ml061730) and spectrophotometrically measured (Shimadzu, model UV-1800, Tokyo, Japan). ML Bio and Jiancheng Bioengineering Institute (Nanjing, China) were sources of the ELISA kits. Concentrations of CAT, T-SOD, and T-AOC were expressed in micromoles per milliliter, GST and GSH-Px as nanograms per milliliter, and MDA as nanomoles per milliliter of serum. Serum concentrations of immunoglobulins, such as IgM, IgA, and IgG, and complement proteins C3 and C4 were determined with the appropriate ELISA kits (WLB-09120, WLB-091301, WLB-050501, E032-1-1, and E033-1-1), respectively, and measured with a microplate reader. The instructions of the manufacturers were stringently followed.

### Intestine sample collection and jejunal morphology analysis

The birds were euthanized with pentobarbital sodium (100 mg/kg BW) intravenously and cut open while maintaining aseptic conditions. For each bird, the organs (magnum, heart, spleen, and liver) were separated and weighed immediately. The weights were expressed as a percentage of their body weight. For each bird, the small intestine samples were processed following an established procedure (Gungor and Erener, 2020). About 3 cm of jejunum were removed and flushed in saline solution to eliminate feed contents, then were immersed in 10% buffered formalin, and were kept under low temperature (4^°^C) for histology analysis. The jejunal tissue sections were embedded in paraffin blocks, and a 6-μm thickness of the tissue was subsequently cut, carefully placed on microscopic glass slides, and stained with a solution of hematoxylin and eosin. For slide examination, a microscope (Olympus BX43 microscope; Olympus Corp., Tokyo, Japan) was employed. To examine the jejunal morphology, 10 intact villi of each selected sample were measured, and the corresponding crypts were selected for measurement and an average value was obtained. The Villi height (VH) was obtained based on the measurement from the tip to the villus-crypt junction of each villus; the villus width (VW) was measured at the middle point of the villus; and crypt depth (CD) was obtained from the basement membrane up to the crypt–villus transition region with the aid of a software (Caseviewer Image). Also, the equation (π × VW × VH) was used to deduce the villi surface area (VSA), while (V/C) was used to obtain the ratio of villi height and crypt depth (Wang et al., 2016; Thiam et al., 2021).

### Egg quality measurement

Following the end of 4, 8, and 12 weeks (a 4-week interval), three eggs per replicate (18 eggs) with a weight close to the range of that replicate were retrieved from each dietary group. The collected eggs were kept under room temperature, and egg quality was determined within 24 h of collection. Upon breakage of each egg, the albumen and the yolk were separated with the aid of an egg separator, and the weight of each was recorded. To measure the thick and thin albumen fractions, the weighed albumen was placed in a 60-mesh sieve at a time bound of 30 s, the thick portion of the albumen was glued to the sieve while the thin portion passed through the sieve as filtrate, and the corresponding weight of each fraction was recorded (Zhou et al., 2021). The eggshells were cleaned to remove any albumen fragments and then naturally dried for 48 h, and the weight was obtained. The proportion of the shell, the yolk, and the albumen relative to egg weight was expressed as shell or albumen or yolk weight/egg weight × 100 (Sarlak et al., 2021). The assessment of egg quality parameters, albumen height, Haugh Unit, and yolk color, was performed with an automatic egg analyzer (ORKA Food Technology Ltd., Ramat HaSharon, Israel). The eggshell breaking strength and eggshell thickness, which is expressed as an average measurement of three points (air cell, equator, and sharp end) (Mwaniki et al., 2018), were examined, respectively, with Egg Force Reader and Eggshell Thickness Gauge (ESTG-1, ORKA Technology Ltd., Ramat HaSharon, Israel).

### Apparent fecal amino acid digestibility

At the end of the experiment (12th week), on a replicate basis, three birds were selected and kept in a cage fitted with a tray for the collection of fecal samples, and this lasted for 3 days. The fecal sample collection was done at an interval of 12 h, and the samples were kept at −20^°^C in tight-closed bags. At the point of collection, it was ensured that all external components, such as feed, feathers, and any other substances, were thoroughly removed from the samples to avoid contamination. The collected feces samples were thawed, weighed, and oven dried for 72 h at 65^°^C, after which it was broken and pulverized into a fine powder that can be sieved through a 0.05 mm mesh. For each metabolic cage, the feed intake and feces weight (dry matter basis) were recorded and used to determine the apparent fecal amino acid digestibility. The feed and fecal samples were further processed for amino acid analysis with HPLC while adopting the method proposed by Varzaru et al. (2013). The apparent fecal amino acid digestibility coefficient was computed; 1 − (amino acid concentration in feces × feces weight) ÷ (amino acid concentration in feed × feed intake) × 100%.

### Statistical analysis

The experiment consists of four groups with six replications, each in a completely randomized design to ensure random allocation of birds to treatments. All the data generated in this study were subjected to a one-way analysis of variance (ANOVA), which ensures that there is no biasness with respect to data normality and equality of variance assumptions (Nwachukwu et al., 2021). Replicates were used as experimental units, and the data were presented as mean and pooled SEM, while the level of significance was considered at a *p*-value < 0.05. Duncan’s multiple range test was employed for *post hoc* comparison to ascertain the variations among the treatment groups. The statistical package used was SPSS software, version 17.0 (SPSS Inc., Chicago, II, United States) (Wang L. et al., 2020).

## Results

### Laying performance

At 4-week intervals throughout the trial, the production performance indices (egg weight, egg mass, hen-day production, mortality and damaged egg rates, feed intake, and feed conversion) were analyzed for all groups. Table 2 presents the outcomes.

**TABLE 2:** Effects of *Clostridium butyricum* and *Brevibacillus* sp. on the performance of laying hens.

Items	Control	CB-z	CB-l	BB-zl	SEM	*P*-value
**Week 1–4**						
AEW (g)	61.41	61.40	60.45	61.06	0.93	0.340
Egg mass (g)	58.30^ab^	59.71^a^	57.11^b^	58.57^ab^	0.99	0.010
HDP%	95.12^b^	97.17^a^	94.49^b^	95.93^ab^	1.18	0.020
Damaged eggs(%)	0.02	0.00	0.03	0.04	0.03	0.150
Mortality(%)	0.01	0.00	0.00	0.00	0.00	0.410
Feed intake (g)	116.58	120.51	119.95	118.78	4.26	0.610
FCR	2.00	2.02	2.10	2.03	0.08	0.271
**Week 5–8**						
AEG (g)	61.84^b^	63.83^a^	61.79^b^	61.44^b^	0.74	0.010
Egg mass (g)	57.63^b^	61.98^a^	58.86^b^	57.82^b^	1.39	0.010
HDP(%)	93.34^b^	97.18^a^	95.34^ab^	94.10^ab^	2.16	0.080
Damaged eggs(%)	0.05	0.05	0.01	0.10	0.05	0.130
Mortality(%)	0.02	0.00	0.00	0.00	0.01	0.090
Feed intake (g)	112.82	114.35	113.00	115.18	4.17	0.810
FCR	1.97^a^	1.84^b^	1.92^ab^	1.90^a^	0.06	0.010
**Week 9–12**						
AEG (g)	59.98^b^	62.06^a^	60.46^b^	60.53^b^	0.96	0.030
Egg mass (g)	53.85^b^	58.45^a^	56.18^ab^	56.26^ab^	1.92	0.020
HDP%	89.76^b^	94.30^a^	92.96^ab^	92.88^ab^	2.74	0.110
Damaged eggs(%)	0.02	0.05	0.08	0.10	0.06	0.240
Mortality(%)	0.01	0.00	0.00	0.00	0.00	0.410
Feed intake (g)	121.17^b^	129.68^a^	126.98^ab^	128.03^a^	4.14	0.050
FCR	2.25	2.22	2.26	2.28	0.09	0.801
**Week 1–12**						
AEG (g)	61.06^b^	62.43^a^	60.95^b^	60.63^b^	0.73	0.012
Egg mass (g)	56.60^b^	60.04^a^	57.38^b^	57.55^b^	1.22	0.002
HDP%	92.73^b^	96.23^a^	94.26^ab^	94.30^ab^	1.65	0.034
Damaged eggs (%)	0.09	0.03	0.09	0.01	0.04	0.144
Mortality(%)	0.01	0.00	0.00	0.00	0.00	0.089
Feed intake (g)	116.87	121.51	119.98	120.66	3.00	0.219
FCR	2.07^ab^	2.02^b^	2.09^a^	2.10^a^	0.05	0.098

CB-z, Clostridium butyricum zlc-17; CB-l, Clostridium butyricum lwc-13; BB-zl, Brevibacillus zlb-z1; SEM, standard error of mean. Data represent the mean of six replicates of three hen each.

^a,b,c^Means within a row with different superscripts differ significantly (*P* < 0.05).

Egg weight was not influenced (*P* ≤ 0.05) by diets at the end of 4 weeks, but CB-z improved egg weight (*P* ≤ 0.05) at weeks 8 and 12, while egg weights for other groups did not vary (*P* ≥ 0.05) with control. Egg mass was increased (*P* ≤ 0.05) by dietary CB-z compared to control and other treatment groups at weeks 4, 8, and 12. Furthermore, at all sampling points, egg mass from the CB group had the highest value, while that of the *Brevibacillus* group was not significant (*P* ≥ 0.05) from the control. A significant increase (*P* ≤ 0.05) in egg production rate due to dietary treatments was observed throughout the feeding period. The egg production rate of the CB-z group was 4.7% higher (*P* ≤ 0.05) and varied from control and other treatment groups, while the *Brevibacillus* group had the lowest egg production rate among the treatment groups throughout the feeding trial. The egg production rate of the CB-l and *Brevibacillus* groups was not significant (*P*≥ 0.05) from control at all measuring points but numerically higher. Zero mortality rate was noticed in the probiotic-based groups but not in the control group, and damaged eggs were not influenced (*P* ≥ 0.05) by diets throughout the study period. Dietary influence on feed intake was not significant (*P* ≥ 0.05) at weeks 4 and 8 but was significant (*P* ≤ 0.05) at week 12. However, there was no variation (*P* ≥ 0.05) among the probiotic-based group. The FCR was enhanced (*P* ≤ 0.05) due to dietary influence at weeks 8 and 1–12 but not at other measuring points. Among the probiotic-based groups, the CB-z group recorded the lowest value, while no variation (*P* ≥ 0.05) between control and other treatments was found.

### Egg quality assessment

The results of egg quality determination are presented in Table 3. The relative weight of the albumen and the yolk differed statistically (*P* ≤ 0.05) between the control group and the dietary group throughout the feeding period, while the relative shell weight was only significant (*P* ≤ 0.05) at week 4 but not at other measuring points. The relative albumen weight consistently increased (*P* ≤ 0.05) due to the treatment effect at all measuring points, except week 4, while the relative yolk weight of the control group increased significantly compared to other treatments throughout the study. There was no variation (*P* ≥ 0.05) in relative albumen weight among the dietary groups during week 12, but the *Brevibacillus* group recorded the least and highest weight at weeks 4 and 12, respectively. Eggshell thickness was improved (*P* ≤ 0.05) by dietary probiotics at weeks 4 and 8, but no variation (*P* ≥ 0.05) was found at week 12. Eggshell strength and yolk color were not improved (*P* ≥ 0.05) by dietary treatments at weeks 4 and 8 but improved (≤ 0.05) by week 12. Eggshell strength did not vary (*P* ≥ 0.05) among the probiotic-based groups, while only the CB group differed from the control for yolk color. Haugh unit and albumen height were not influenced (*P* ≥ 0.05) by dietary probiotics at the end of week 4, but significant improvements (*P* ≤ 0.05) due to treatment effect were notable at the end of weeks 8 and 12. The Haugh unit and the albumen height of the probiotic-based groups were consistently higher (*P* ≤ 0.05) compared to the control. Among treatments, no significant variation (*P* ≥ 0.05) in the albumen height was found at the end of week 8, but the CB group was greater (*P* ≤ 0.05) than the *Brevibacillus* group at the end of week 12. Similarly, at the end of weeks 8 and 12, no statistical differences (*P* ≥ 0.05) were found for the Haugh unit among the treatment groups. Thick-to-thin albumen ratio was consistently significant (*P* ≤ 0.05) throughout the entire experiment. At the end of weeks 4 and 8, only the CB-z group and the *Brevibacillus* group were, respectively, significant (*P* ≤ 0.05) from the control, while all the treatment groups were significant from control at week 12, and no observable variations (*P* ≥ 0.05) among treatments were found.

**TABLE 3:** Effects of *Clostridium butyricum* and *Brevibacillus* sp. on the egg quality of laying hens.

Items	Control	CB-z	CB-l	BB-zl	SEM	*P*-value
**Week 4**						
Relative albumen weight(%)	59.58^b^	61.06^ab^	59.98^b^	62.47^a^	1.37	0.020
Relative yolk weight(%)	28.48^a^	27.30^ab^	28.03^a^	26.77^b^	0.85	0.030
Relative shell weight(%)	10.83^a^	10.29^b^	10.81^a^	10.17^b^	0.34	0.010
Shell thickness (mm)	46.88^b^	48.59^a^	45.70^b^	46.49^b^	0.93	0.010
Shell strength, (*N*)	45.40	46.28	43.44	46.61	3.61	0.540
Yolk color	7.11	7.17	7.06	7.22	0.50	0.960
Albumen height (mm)	7.80^b^	8.93^a^	8.33^ab^	8.94^a^	0.50	0.010
Haugh units	86.95^b^	93.27^a^	89.40^ab^	93.46^a^	3.15	0.030
Thick to thin albumen ratio	1.08^b^	1.87^a^	1.14^b^	1.35^b^	0.22	0.010
**Week 8**						
Relative albumen weight(%)	59.58^ab^	61.23^a^	57.64^bc^	56.06^c^	1.75	0.010
Relative yolk weight(%)	29.01^a^	26.61^b^	30.31^a^	30.00^a^	1.07	0.010
Relative shell weight(%)	10.74	11.07	10.93	11.12	0.58	0.770
Shell thickness (mm)	44.88^ab^	47.42^a^	41.07^b^	45.46^a^	2.60	0.020
Shell strength, *N*	40.61	44.27	41.17	37.79	4.50	0.260
Yolk color	5.72	8.50	8.72	5.67	3.38	0.540
Albumen height (mm)	6.81	7.12	7.07	6.90	0.64	0.870
Haugh units	80.23	81.09	81.42	76.42	6.02	0.620
Thick to thin albumen ratio	1.00^b^	1.09^ab^	1.18^ab^	1.36^a^	0.20	0.080
**Week 12**						
Relative albumen weight(%)	58.24^b^	63.43^a^	64.02^a^	62.74^a^	1.41	0.010
Relative yolk weight(%)	31.48^a^	25.56^b^	26.32^b^	25.80^b^	1.15	0.010
Relative shell weight(%)	10.31	10.26	10.13	10.18	0.44	0.932
Shell thickness (mm)	44.33	45.88	44.77	44.35	1.23	0.235
Shell strength, *N*	36.97^ab^	39.05^b^	40.43^ab^	41.21^a^	2.66	0.020
Yolk color	5.72	7.17	6.22	6.94	0.53	0.010
Albumen height (mm)	6.19^c^	7.92^a^	7.90^a^	7.22^b^	0.35	0.020
Haugh units	76.16^b^	87.36^a^	84.75^a^	84.29^a^	4.34	0.010
Thick to thin albumen ratio	1.14^b^	1.52^a^	1.54^a^	1.43^a^	0.16	0.020

CB-z, Clostridium butyricum zlc-17; CB-l, Clostridium butyricum lwc-13; BB-zl, Brevibacillus zlb-z1; SEM, standard error of mean. Data represent the mean of six replicates of three hen each.

^a,b,c^Means within a row with different superscripts differ significantly (*P* < 0.05).

### Hematological and serum biochemical profiles

The hematological indices of laying hens fed dietary *C. butyricum* and *Brevibacillus* sp. are presented in Table 4. The blood indices such as WBC, MCV, MCH, PLT, heterophils, neutrophils, and H/L were influenced (*P* ≤ 0.05) by treatments, while other indices such as eosinophils, RBC, Hb, monocytes, PCV, and MCHC were not affected (*P* ≥ 0.05) by diets. Also, WBC count, heterophils, and lymphocytes were not significantly different (*P* ≥ 0.05) among the treatment groups. Only birds in the CB-z group recorded a lower H/L ratio among the supplemented groups with a level of significance (*P* ≤ 0.05) compared to the control group.

**TABLE 4:** Effects of *Clostridium butyricum* and *Brevibacillus* sp. on the hematological indices of laying hens.

Items	Control	CB-z	CB-l	BB-zl	SEM	*P*-value
WBC (× 10^9^/L)	12.10^b^	17.22^a^	18.45^a^	18.51^a^	1.42	0.010
RBC (× 10^12^/L)	2.26	2.30	2.25	2.23	0.12	0.829
Hb (g/L)	71.00	73.80	73.67	73.16	5.02	0.837
PCV (%)	35.00	35.00	34.68	35.16	1.98	0.991
MCV (fL)	155.00^b^	151.86^ab^	156.11^ab^	157.42^a^	3.45	0.048
MCH (Pg)	31.40^b^	30.28^ab^	33.36^a^	32.77^a^	1.41	0.055
MCHC (g/L)	203.00	199.40	213.67	208.00	7.38	0.164
Platelets (× 10^9^/L)	11.70^a^	8.40^b^	11.67^a^	9.67^ab^	2.04	0.088
Heterophil (× 10^9^/L)	6.24^ab^	7.63^ab^	9.69^a^	8.90^b^	1.81	0.056
Lymphocytes (× 10^9^/L)	5.18^ab^	7.65^a^	6.78^ab^	6.51^b^	1.47	0.086
H/L	1.20^b^	0.99^c^	1.42^a^	1.36^a^	0.18	0.036
Monocyte (× 10^9^/L)	0.16	0.59	0.40	0.44	0.27	0.202
Eosinophil (× 10^9^/L)	0.05	0.17	0.13	0.39	0.24	0.332
Basophil (× 10^9^/L)	0.98^b^	0.95^b^	1.44^ab^	2.28^a^	0.83	0.094

CB-z, Clostridium butyricum zlc-17; CB-l, Clostridium butyricum lwc-13; BB-zl, Brevibacillus zlb-z1; SEM, standard error of mean; WBC, white blood cells count; RBC, red blood cells count; Hb, hemoglobin count; PCV, packed cell volume; MCV, mean corpuscular volume; MCH, mean corpuscular hemoglobin; MCHC, mean corpuscular hemoglobin concentration; H/L, heterophil/lymphocyte.

Data represent the mean of six replicates of three hen each.

^a,b,c^Means within a row with different superscripts differ significantly (*P* < 0.05).

The influence of dietary *C*. *butyricum* and *Brevibacillus* on the serum index, immunity and oxidant and antioxidant parameters, of laying hens is presented in Table 5. Serum MDA was significantly higher (*P* ≤ 0.05) in the control than in the treatment group, whereas the *Brevibacillus* group recorded the lowest concentration of the MDA content in the serum. The concentrations of the antioxidant enzymes T-SOD, GST, GSH-Px, and CAT were increased (*P* ≤ 0.05) due to dietary treatments, whereas T-AOC was not influenced (*P* ≥ 0.05) by the diets. The concentrations of serum IgM and IgA were influenced (*P* ≤ 0.05) by diets, but no variations (*P* ≥ 0.05) in IgG due to the dietary treatment were observed. The *C. butyricum* group had the highest immunoglobulin concentrations compared to the *Brevibacillus* group. Dietary treatments influenced (*P* ≤ 0.05) complement protein C3 (*P* ≤ 0.05) but not C4.

**TABLE 5:** Effects of *Clostridium butyricum* and *Brevibacillus* sp. on the serum antioxidant and immune capacity of laying hens.

Items	Control	CB-z	CB-l	BB-zl	SEM	*P*-value
MDA (nmol/mL)	8.54^a^	5.00^b^	4.40^b^	2.79^c^	1.01	0.010
CAT (U/mL)	9.67^c^	13.34^b^	13.08^b^	14.83^a^	0.27	0.012
T-SOD (U/mL)	107.41^c^	143.81^a^	114.76^c^	130.22^b^	4.80	0.010
T-AOC (U/mL)	11.50	13.17	12.67	14.30	2.43	0.370
GSH-Px (ng/mL)	53.45^b^	76.53^a^	51.86^b^	68.45^a^	7.78	0.010
GST (ng/mL)	16.57^c^	18.93^ab^	19.32^a^	18.52^b^	0.55	0.010
IgG (μg/mL)	58.33	65.65	61.47	65.12	8.14	0.486
IgM (ng/mL)	2335.50^b^	3325.00^a^	3343.33^a^	2372.50^b^	581.51	0.012
IgA (ng/mL)	4762.78^c^	6104.44^a^	5990.55^a^	5512.83^b^	241.80	0.010
C3 (mg/mL)	0.073^b^	0.085^a^	0.088^a^	0.075^b^	0.006	0.010
C4 (mg/mL)	0.044	0.048	0.045	0.046	0.002	0.432

CB-z, Clostridium butyricum zlc-17; CB-l, Clostridium butyricum lwc-13; BB-zl, Brevibacillus zlb-z1; SEM, standard error of mean. MDA-malondialdehyde, CAT-catalase, T-SOD- total superoxide dismutase, T-AOC-total antioxidant capacity, GSH-Px-glutathione peroxidase, GST-glutathione transferase, IgG-immunoglobulin G, IgM- immunoglobulin M, IgA- immunoglobulin A, C3 and C4-complement proteins. Data represent the mean of six replicates of three hen each.

^a,b,c^Means within a row with different superscripts differ significantly (*P* < 0.05), SEM: standard error of mean.

### Apparent fecal amino acid digestibility

Table 6 presents data on the apparent fecal amino acid digestibility of laying hens fed probiotics-based diets. There was a significant improvement (*P* ≤ 0.05) in the digestibility of essential amino acids (isoleucine, valine, leucine, methionine, histidine lysine, and phenylalanine) and non-essential amino acids (glycine, serine, methionine-cysteine, and tyrosine) and crude protein, due to influence of diets. Other amino acids including asparagine, threonine, glutamic acid, proline, alanine, cysteine, lysine, arginine, and tryptophan were not influenced (*P* ≥ 0.05) by treatments. Among the diet group, the CB-z group had the highest value for digestibility of all amino acids. No variations (*P* ≥ 0.05) exist between the *Clostridium groups* for all amino acid digestibility coefficients, while the *Brevibacillus* group differed in methionine-cysteine and serine from the *C. butyricum* group.

**TABLE 6:** Effects of *Clostridium butyricum* and *Brevibacillus* sp. on the apparent fecal amino acid digestibility coefficient of laying hens.

Apparent digestibility(%)	Control	CB-z	CB-l	BB-zl	SEM	*P*-value
Crude protein	59.20^c^	70.64^ab^	73.29^a^	64.80^bc^	5.37	0.005
Asparagine	75.29	81.53	80.60	75.80	4.25	0.104
Threonine	69.48	76.95	75.33	68,76	5.41	0.102
Serine	77.63^ab^	83.24^a^	80.94^ab^	76.21^c^	4.24	0.048
Glutamic acid	84.57	88.96	87.56	84.75	2.97	0.120
Proline	80.42	84.80	84.33	82.49	3.16	0.185
Glycine	-3.14^c^	25.75^a^	31.09^a^	8.74^b^	16.70	0.025
Alanine	65.77	73.41	73.09	66.22	5.64	0.107
Cysteine	73.58	80.01	78.15	72.77	4.60	0.103
Valine	73.91	79.92	79.76	74.97	4.12	0.090
Methionine	83.01	87.55	86.25	82.74	3.05	0.092
Met + Cys	79.28^ab^	84.57^a^	83.05^ab^	78.80^b^	3.62	0.093
Isoleucine	74.16^b^	81.48^a^	80.81^a^	76.13^ab^	4.34	0.060
Leucine	80.21^c^	85.84^a^	84.98^ab^	80.94^bc^	3.13	0.036
Tyrosine	80.54^c^	88.88^a^	87.53^ab^	82.59^bc^	3.60	0.010
Phenylalanine	89.25^b^	94.66^a^	94.39^a^	93.55^a^	2.45	0.012
Histidine	51.02^b^	63.59^a^	68.79^a^	61.88^ab^	8.00	0.023
Lysine	73.19^b^	80.23^a^	78.92^ab^	74.75^ab^	4.52	0.048
Arginine	83.97	87.84	87.08	84.41	2.67	0.112
Tryptophan	73.55	77.22	75.92	76.32	4.63	0.665

CB-z, Clostridium butyricum zlc-17; CB-l, Clostridium butyricum lwc-13; BB-zl, Brevibacillus zlb-z1; SEM, standard error of mean; Met + cys, methionine cysteine. Data represent the mean of six replicates of three hen each.

^a,b,c^Means within a row with different superscripts differ significantly (*P* < 0.05).

### Relative organ weight and jejunal villi morphological structure

The organ weights of the magnum, the spleen, the heart, and the liver (expressed as relative weight) of laying hens were fed dietary *C. butyricum* and *Brevibacillus*, which are listed in Table 7. The relative weights of the magnum, the heart, and the liver were not statistically different (*P* ≥ 0.05) from the control, although the weight of the spleen was increased (*P* ≤ 0.05) due to dietary influence.

**TABLE 7:** Effects of *Clostridium butyricum* and *Brevibacillus* sp. on the organ index of laying hens.

Items	Control	CB-z	CB-l	BB-zl	SEM	*P*-value
Magnum	2.01	2.00	1.78	1.84	0.25	0.352
Spleen	0.09^b^	0.09^ab^	0.11^a^	0.08^b^	0.01	0.040
Heart	0.36	0.37	0.35	0.36	0.04	0.790
Liver	1.38	1.65	1.66	1.50	0.20	0.152

CB-z, Clostridium butyricum zlc-17; CB-l, Clostridium butyricum lwc-13; BB-zl, Brevibacillus zlb-z1; SEM, standard error of mean. Data represent the mean of six replicates of three hen each.

^a,b^Means within a row with different superscripts differ significantly (*P* < 0.05).

The morphological characteristics of the jejunal villi of laying hens fed *C. butyricum* and *Brevibacillus* are presented in Table 8. The villous indices (height, width, surface area), crypt depth, and villi height to crypt depth ratio were all influenced (*P* ≤ 0.05) by a probiotic-based diet. Villous height, width, surface area, and villi height to crypt depth were significantly increased (*P* ≤ 0.05), whereas crypt depth was reduced in the treatment group compared to the control. All villi morphometrics differed (*P* ≤ 0.05) among the treatments. The CB-z group recorded the highest value for villous surface area, height, and width, while the *Brevibacillus* group had the least value for crypt depth and the highest value for villi height to crypt depth ratio.

**TABLE 8:** Effects of *Clostridium butyricum* and *Brevibacillus* sp. on the jejunal villi morphometry of laying hens.

Items	Control	CB-z	CB-l	BB-zl	SEM	*P*-value
VH (μm)	938.80^c^	1347.65^a^	1186.63^b^	1294.05^ab^	36.18	0.010
VW (μm)	141.42^d^	208.15^a^	167.60^c^	188.71^b^	5.66	0.012
CD (μm)	155.60^a^	113.40^bc^	123.80^b^	98.83^c^	5.45	0.018
VH:CD	6.35^c^	12.08^ab^	9.75^b^	13.85^a^	0.75	0.010
VSA (mmm^3^)	0.44^d^	0.88^a^	0.63^c^	0.77^b^	0.03	0.010

CB-z, Clostridium butyricum zlc-17; CB-l, Clostridium butyricum lwc-13; BB-zl, Brevibacillus zlb-z1; SEM, standard error of mean; VH-villi height, VW-villi width, CD-crypt depth, VH:CD, villi height to crypt depth ratio, VSA, villi surface area. Data represent the mean of six replicates of three hen each.

^a,b,c,d^Means within a row with different superscripts differ significantly (*P* < 0.05), SEM, standard error of mean.

## Discussion

Eggs are very important to consumers due to their high nutrient and biological quality; therefore, feeding laying hens with diets supplemented with natural feed additives, such as probiotics, can provide results that are vital for the commercial laying hens industry. The spores of *Clostridium butyricum* and *Brevibacillus* were used in the present study due to their stability in the gut and thus are regarded as safe use for poultry nutrition. Dietary supplementation with *Clostridium butyricum* and *Brevibacillus* sp. had no negative impact on the laying hens throughout the feeding period. This lends evidence that the newly designed strains of *C. butyricum* and *Brevibacillus* are suitable for peak-laying hens. They could also be used as a safe feed additive in the poultry industry as reported previously with other probiotic strains (Wei et al., 2020; Yang et al., 2020; Ahmat et al., 2021; César et al., 2022). There is a dearth of evidence on the influence of *Brevibacillus* sp. in the diet of laying hens, and *Bacillus* sp. would be used for comparison.

### Laying performance

Improvement in egg production rate and egg weight is of critical economic value to the poultry industry. Probiotics, including *Clostridium butyricum* (Xiang et al., 2019; Zhan et al., 2019; Wang W. W. et al., 2020; Wang Y. et al., 2021) and *Bacillus* sp., *B. velezensis* (Wei et al., 2020), *B. subtilis* (Guo et al., 2017; Darsi and Zhaghari, 2021; Souza et al., 2021), and *B. licheniformis* (Pan et al., 2022), were reported to enhance egg production in laying hens. Also, combined *Bacillus* strains caused an 8% improvement in laying performance compared to the control (Yang et al., 2020). The increased egg production rate could be due to an improvement in nutrient utilization (Souza et al., 2021), which is accrued to a positive effect of probiotics on the beneficial gut microbial population (Xu et al., 2022) and gut morphology (Song et al., 2019). In the current study, our findings revealed that *Clostridium butyricum* and *Brevibacillus* significantly improved egg production rates. The improvement could be adducible to better nutrient utilization orchestrated by a decrease in stress response, enhanced gut health, and improved immune function, as observed in this study. This claim is supported by previous studies in laying hens, which demonstrated that probiotic-induced improvement in physiological indices, including immunity index, intestinal morphology, and antioxidant capacity culminated in significantly increased laying performance (Deng et al., 2021; Wang Y. et al., 2021; Pan et al., 2022). However, some studies found no probiotic-induced effect on egg production (Shi et al., 2020; Wang J. et al., 2021; Ray et al., 2022). The discrepancies may be due to the age of laying hens and probiotics composition and dosage. Enhanced laying performance often reflects egg mass output and egg weight. Our findings revealed a significant improvement in egg weight and egg mass in response to dietary probiotics, and this is in line with previous reports that probiotics exerted a beneficial effect on egg weight (Zhan et al., 2019; Wang Y. et al., 2021; Ray et al., 2022). The improvement suggests that probiotics are natural growth enhancers through efficient nutrient utilization. Nevertheless, some studies reported a non-significant influence of probiotics on egg weight (Xiang et al., 2019; Darsi and Zhaghari, 2021; Souza et al., 2021). Feed consumption (FI) and FCR are often used as indicators for feed utilization efficiency. In previous reports, probiotics including *Clostridium butyricum* (Xiang et al., 2019; Wang W. W. et al., 2020; Wang Y. et al., 2021), *Bacillus* sp. (Neijat et al., 2019; Yang et al., 2020; Pan et al., 2022), and combined probiotics strains (Ray et al., 2022; Xu et al., 2022) enhanced feed efficiency in laying hens. Similarly, in this study, *Clostridium butyricum* improved feed utilization, and consequently, the enhanced feed efficiency was a boost to the production potential of the animal. The improvement in feed efficiency may be the capacity of probiotics to enhance gut morphology and health *via* increased villi height for better nutrient absorption (Wang W. W. et al., 2020; Pan et al., 2022) and the production of bacteriocins and volatile bacteriostatic substances, which can suppress pathogen invasion (Chen et al., 2007). Conversely, there was no influence of probiotics on FCR (Upadhaya et al., 2019) and feed intake (Neijat et al., 2019). This may be due to the inclusion level of probiotics in the diets. Furthermore, in this study, the probiotic-based group recorded zero mortality rate relative to the control. In line with the current findings, Xiang et al. (2019) reported zero mortality rate in laying hens fed *Clostridium butyricum.* The zero-mortality observed in the diet group could be that continuous feeding of probiotics enhanced the health status of the birds as evidenced by gut integrity and better antioxidant and immune function. Conclusively, the improved egg weight, egg production rate, feed utilization efficiency, and egg mass are suggestive of the positive response of laying hens to dietary probiotics supplementation.

### Egg quality

Maintenance of external (shell quality) and internal (albumen and yolk) components of the egg is of utmost priority in the laying hen’s industry, to gain consumers’ acceptance while meeting up with market demands. In the study, our findings showed improvement in eggshell thickness and eggshell strength in response to dietary probiotics. This corroborates the previous findings that supplementation of probiotics: *B. subtilis* PB6 in broiler breeder hens (Darsi and Zhaghari, 2021; Souza et al., 2021), *B. subtilis* (Guo et al., 2017; Fathi et al., 2018), and BLCC1-0238 (Upadhaya et al., 2019) in laying hens improved eggshell thickness. Also, *Clostridium butyricum* exerted a beneficial effect on eggshell thickness and eggshell strength (Zhan et al., 2019; Wang Y. et al., 2021). Probiotics have been found to enhance the growth of beneficial bacteria, and the proliferation of these microbes enhances the fermentation rate, which culminates in the accumulation of short-chain fatty acids (SCFAs) and a reduction in luminal pH (Forte et al., 2016). Also, the SCFAs enhance the growth and nourishment of intestinal villi structures for a better absorption rate (Zou et al., 2019). Thus, improvement in eggshell quality could be linked to the capacity of probiotics to increase the assimilation and retention levels of calcium in the serum of laying birds (Zhan et al., 2019; Attia et al., 2020), which would facilitate calcium deposition on the shell glands. Conversely, Wang W. W. et al. (2020) demonstrated that *Clostridium butyricum* had no influence on eggshell thickness and strength, while B. *subtilis* had no effect on eggshell thickness but enhanced eggshell strength (Upadhaya et al., 2019). These variations may be due to age and the laying phase of the hens. In addition, albumen quality is of great importance for the food processing and health industry, which utilizes the albumen as raw materials for further production of foods and drugs. Pieces of evidence demonstrated that *Bacillus subtilis* (Chen et al., 2019; Neijat et al., 2019; Yang et al., 2020) and *B. velezensis* (Wei et al., 2020) improved HU and albumen height in broiler breeders and laying hens. In another study, *Bacillus* strains improved HU and the protein index in laying hens (Mazanko et al., 2018). Also, *Clostridium butyricum* was also found to enhance albumen height (Wang W. W. et al., 2020; Wang Y. et al., 2021) and albumen crude protein (Xiang et al., 2019). The improvement in albumen quality due to dietary probiotics effect may be accrued to enhanced nutrient digestibility, which would improve protein synthesis. It has been reported that probiotics stimulate the activities of digestive enzymes that cause a resultant increase in nutrient utilization and protein digestibility (Ahiwe et al., 2020). In the present study, we observed a similar distinct increase in albumen indices (albumen height, Haugh unit, and thick-to-thin albumen ratio) in response to dietary probiotics. The current study, to the best of our knowledge, would be the first to report the influence of probiotics on the thick-to-thin albumen ratio. The improvement in albumen quality may reflect an increase in protein synthesis (Lei et al., 2013). The enhanced HU values could be accrued to better bioavailability of nutrients and better gross digestible energy due to probiotics. Also, the capacity of *Clostridium butyricum* and *Brevibacillus* to modulate the microflora composition in the body of an organism may have led to a beneficial effect on oviduct flora with consequent improvement in albumen synthesis. This claim is supported by the study of Camarda et al. (2000), which demonstrated that some pathogens may colonize the oviduct and impair its functions. The improvement in albumen quality indices suggests the production of high-quality eggs with better albumen viscoelasticity and shelf life (Zhang et al., 2020). On the contrary, *Clostridium butyricum* had no effect on albumen height, HU value (Zhan et al., 2019), and HU value (Wang W. W. et al., 2020). The discrepancies may be related to the type of probiotics used. Probiotics including *Clostridium butyricum* (Wang W. W. et al., 2020; Wang Y. et al., 2021) and *Bacillus* sp. (Liu et al., 2019; Zhou et al., 2020) improved yolk color in laying hens. The improvement in the yolk color may be accrued to the probiotic composition. In contrast, we observed no effect of diets on yolk color, similar to the results of Lei et al. (2013), which revealed that the dietary influence of *B. licheniformis* on egg yolk color was not significant. The non-significant effect on yolk color could be a reflection that dietary probiotics do not play a key role in the metabolism of xanthophyll, which is a non-nutritive component. According to the aforementioned research findings, *Clostridium butyricum* and *Brevibacillus* could be employed as secure feed additives in laying hen diets to enhance albumen and eggshell quality and two factors that are economically important and beneficial to the poultry sector.

### Hematological indices, antioxidant capacity, and immune function

Farm animals are susceptible to oxidative stress, which often cause decreased immune function. Most often, blood parameters are used to investigate the level of stress in animals. In the present study, blood indices (WB, PLT, neutrophil, lymphocytes, basophils, H/L) were influenced by dietary probiotics. There are reports that *Bacillus subtilis* had no influence on blood WBC, RBC, or lymphocytes in laying hens (Shi et al., 2020) and broiler chickens (Park et al., 2018). In this study, the decreased H/L ratio is similar to the findings of César et al. (2022), and the decreased H/L ratio could be due to the immunomodulatory effect of the dietary bioactive components that can also suppress pathogenic conditions in poultry (Kogut and Klasing, 2009). The decrease in the H/L ratio due to dietary probiotics suggests that *Clostridium butyricum* and *Brevibacillus* could stabilize the health status of the laying hens. Therefore, the birds’ welfare can be improved under farm conditions, since probiotic supplementation in the diet of laying hens can be considered a dietary strategy for stress reduction in laying hens.

When there is a disequilibrium between the antioxidant system and reactive oxygen species (ROS) as output, oxidative stress becomes the norm, and the homeostatic balance of the animals is disrupted (Surai et al., 2019). Oxidative stress may impair reproductive performance because it induces the synthesis of ROS, which could disintegrate proteins and nucleic acid, and culminates in tissue damage (Pisoschi et al., 2021). There are pieces of evidence that probiotics could mask the negative effects of oxidative stress, enhance the activities of antioxidative enzymes and dietary CB; improved the activities of GSH-Px, CAT, and T-SOD with a concomitant decrease in the MDA content of the serum (Zhan et al., 2019), reduced the MDA content in the intestine (Xiang et al., 2019), improved serum antioxidant status but decreased T-AOC (Wang Y. et al., 2021) in laying hens. Also, Zhou et al. (2020) revealed that laying hens fed diets supplemented with *B. amyloliquefaciens* BLCC1-0238 had enhanced GSH-Px and GST activities but no effect was notable on the serum concentrations of the antioxidant system (CAT, T-AOC, T-SOD) and the MDA content. The increased antioxidant capacity could probably be due to the capacity of probiotics to increase the activities of antioxidant enzymes while the reduced T-AOC could be due to less occurrence of ROS in the body system. Our present findings revealed that *C. butyricum and Brevibacillus* enhanced the activities of GSH-Px, GST, CAT, and T-SOD and had no effect on T-AOC while reducing the MDA content. This lends evidence once again that probiotics including *C. butyricum and Brevibacillus* could modulate the antioxidant capacity of laying hens. An indication that the probiotics as feed additives could act as a boost to the antioxidant capacity of the host while lipid peroxidation activity is reduced. The enhanced antioxidant system may be due to the capacity of CB to synthesize butyrate and H2, which scavenge ROS and improve the activity of the antioxidative enzymes (Jahns et al., 2015). The lack of probiotic-diet effect on T-AOC could be that the body may be insensitive to such probiotic-mediated responses. All together, these findings indicate that *C. butyricum* and *Brevibacillus* could mitigate oxidative stress in laying hens through the stimulation of enzymatic components, which in turn supports better egg production rate and egg quality. An indication that these probiotics with antioxidant effects could be utilized in the laying industry as probiotic antioxidants.

The immunity index of animals is often measured based on the serum concentrations of immunoglobulins (IgM, IgA, and IgG) due to their key role in immune regulation and disease resistance (Mountzouris et al., 2010). Previous reports demonstrated that *B. amyloliquefaciens* significantly increased serum concentrations of IgG and IgA (Ahmat et al., 2021) and the probiotic complex (Deng et al., 2021), and *B. subtilis* (Qiu et al., 2021) enhanced the serum immunoglobulins of IgG, IgA, and IgM in broiler birds. There are pieces of evidence that *B. amyloliquefaciens* increased IgG and IgA levels (Zhou et al., 2020) and CB enhanced IgA, IgY, and IgM (Zhan et al., 2019) in laying hens. In a similar vein, our findings demonstrated that concentrations of the immunoglobulins (IgA and IgM) in the serum were significantly increased, while no effect was notable for IgG. Probiotics have been found to reduce the colonization of intestinal pathogens and stimulate the synthesis of natural antibodies (Haghighi et al., 2006). Thus, the improved immunoglobulin synthesis may be associated with the capacity of probiotics to exert immunomodulatory effects. Increased bioavailability of amino acids is critical to the synthesis of immunoglobulins (Azzam et al., 2015); therefore, the significant improvement may be adducible to improved digestibility of amino acids. Also, the small intestine acts as an immune protection barrier in animals (Patterson and Burkholder, 2003), and the improved gut integrity may favor the activities of intestinal mucosal cytokines, thereby enhancing the immune status of the birds. The immunity status of animals is often linked with the animal’s capacity to counteract oxidative stress *via* enhanced antioxidant capacity (Wan et al., 2018), and the enhanced activities of the antioxidant enzymes may be a contributory factor. In one study, *B. subtilis* C-3102 enhanced IgM concentration linearly but had no effect on serum concentrations of IgA and IgG (Liu et al., 2019). The discrepancies in the studies could be the environmental hygiene and the type of probiotics used. Complement proteins play key roles in immune function; thus, they are often used to assess the immune status of animals. Dietary supplementation of *C. butyricum* enhanced C3 concentration in broiler chickens (Zhang et al., 2016) and enhanced C3 and C4 concentration in laying hens (Zhan et al., 2019). Further analysis of the immunity index could be measured with the weight of the spleen, probably because the spleen is involved in cellular and humoral immunity of the body. Our findings showed that the relative weight of the spleen was improved with dietary *Clostridium butyricum* and similar to reports in laying hens (Awad et al., 2010; Zhan et al., 2019) and that of broilers (Chen et al., 2013; Ahmat et al., 2021). The aforementioned findings suggest that probiotics as nutritional components in the diets of laying hens can stimulate the local immune system in the gut, although the systemic effects are notable in the blood. The immunomodulatory effect of *Clostridium butyricum* and *Brevibacillus* on laying hens was evidenced in the enhanced level of immunoglobulins and the weight of the spleen. We could deduce that the improved immune status and the activities of antioxidant enzymes contributed immensely to the health status, which supported laying performance and production of eggs with better shell and albumen quality.

### Apparent fecal amino acid digestibility

Data on the digestibility of amino acids using *Clostridium butyricum* and *Brevibacillus* sp. supplemented in laying hens’ feed are scarce. This study demonstrated that dietary probiotics can effectively improve the fecal digestibility of amino acids and crude protein. This study would be the first study to report the influence of probiotics on the apparent fecal digestibility of crude protein and amino acids. The digestibility of crude protein and most essential amino acids (Val, Met, Met-cys, Ile, Leu, Tyr, Phe, His) and non-essential amino acid (Glycine) was significantly high compared to the control. The enhanced digestibility may be due to the capacity of probiotics to positively influence the host by improving the intestinal structures and suppressing pathogens’ proliferation (Emami et al., 2020). It could also be that the probiotics used provided a favorable environment for the degradation of nutrients from feed. There are pieces of evidence that *Clostridium butyricum* could enhance secretion and activities of digestive enzymes (Zhang et al., 2016) and modulate gut microflora (Duan et al., 2018), which in turn could promote nutrient absorption. Also, in laying hens, probiotics enhanced nutrient retention, which acts as a catalyst for improved performance (Neijat et al., 2019). A study reported that in broiler diets supplemented with probiotics, increased ileal digestibility of nutrients leads to a corresponding increased performance (Mountzouris et al., 2010). The enhanced digestibility of crude protein may account for the improvement in albumen synthesis; it has been reported that diets with high crude protein values enhance albumen quality (Shim et al., 2013). Therefore, we deduced that the increased digestibility of amino acids was the basis of enhanced immunoglobulin secretions, laying performance, and egg quality.

### Jejunal villi morphology

Digestion and absorption as key events in the digestive tract occur mainly in the jejunum, which is the part of the small intestine, and significant variations in this region could suggest changes in digestion and absorption capacity across the diets. The villi height, crypt depth, villi width, and surface area all reflect the gut integrity and strength of nutrient absorption capacity. The improved nutrient absorption capacity of the gut is evidenced by decreased crypt depth and increased villi height, villi width, and villi height to crypt depth ratio (Shamoto and Yamauchi, 2000). Whereas, shorter villi and deeper crypts cause less utilization of nutrients (Xu et al., 2020) because the energy needed for the metabolic process is diverted to gut cell renewal in response to normal sloughing or inflammatory response (Giannenas et al., 2014). There are shreds of evidence that *C. butyricum* increased jejunal villi height (Zhang et al., 2011) and ileal villi height (Wang W. W. et al., 2020) and villi height to crypt depth ratio but decreased crypt depth (Xiang et al., 2019) in laying hens. Also, *C. butyricum* increased villi height and villi height to crypt depth ratio but decreased crypt depth in broilers (Zhang et al., 2016; Abdel-Latif et al., 2018). In addition, *Bacillus* sp. increased jejunal villi height and villi height to crypt depth ratio and decreased crypt depth (Yang et al., 2020; Pan et al., 2022) in laying hens. The improvement in gut morphology may be accrued to the capacity of microorganisms present in the gut to extend the length of the intestine (Chen et al., 2016). Our findings are in tandem with the previous findings on enhanced villi height and villi height to crypt depth ratio, broader surface area, and decreased crypt depth due to dietary probiotics. The improved jejunal villi structures could be that *Clostridium butyricum* enhanced the digestion of carbohydrates and the synthesis of SCFAs (Shah et al., 2019), which supply nutrients to the intestinal goblet cells and protect intestinal epithelial cells (Liu et al., 2019; Guo et al., 2021). Conversely, *C. butyricum* had no distinct influence on the jejunum microscopic structures (Wang W. W. et al., 2020). This could be due to probiotic composition, age of laying hens, and duration of feeding. This suggests that the improvement in amino acid absorption probably was facilitated by improved intestinal structures. We could therefore deduce that improved villi structures, which provided larger absorption surface area, were culminated in enhanced utilization of nutrients in the feed and increased amino acid digestibility, which translated into increased laying performance and egg quality.

## Conclusion

*Clostridium butyricum* and *Brevibacillus* spores improved protein synthesis and nutrient utilization while regulating gut function and health status in laying hens. The aforementioned findings revealed that the supplementation of *Clostridium* and *Brevibacillus* spores as probiotics in the diet of laying hens might be a promising safe feed additive and an enhancer for the intestinal health of laying hens. In comparison to other treatments, *Clostridium butyricum* (zlc-17) was more efficient in enhancing egg production rate, feed efficiency, albumen quality, immunoglobulin and antioxidant enzymes, amino acid digestibility, and jejunal villi microscopic structures. Hence, 0.02% of *Clostridium butyricum* (zlc-17) is suitable to be supplemented in the diet of laying hens at the peak phase.

## Data availability statement

The original contributions presented in this study are included in the article/supplementary material, further inquiries can be directed to the corresponding author/s.

## Ethics statement

The animal study was reviewed and approved by the Animal ethics and Use Committee of the Feed Research Institute of the Chinese Academy of Agricultural Sciences, Beijing, China. CAAS. No: 20200507S0600103.

## Author contributions

KQ, T-HS, and S-GW: conceptualization. KQ, X-YC, Y-BS, and UO: resources data. UO: writing – original draft. KQ, JW, H-JZ, T-HS, and S-GW: supervision. KQ and UO: writing and editing. S-GW and G-HQ: funding. All authors reviewed and accepted this final version of the manuscript.

## Abbreviations

ADFI: average feed intake
AEG: average egg weight
AH: albumen height
BB: *Brevibacillus*
CB: *Clostridium butyricum*
CAT: catalase
CD: crypt depth
FCR: feed conversion ratio
GST: glutathione transferase
GSH-Px: glutathione peroxidase
HDP: hen day production
HU: Haugh unit
H/L: heterophil to lymphocyte ratio
IgM: immunoglobulin M
IgG: immunoglobulin G
IgA: immunoglobulin A
MCH: mean corpuscular hemoglobin
MCV: mean corpuscular volume
MDA: malondialdehyde
ROS: reactive oxygen species
RBC: red blood cells count
SCFA: short-chain fatty acid
TAO-C: Total antioxidant capacity
TSOD: Total peroxide dismutase
VH: Villi height
VW: villus width
VSA: villi surface area
V/C: villi height to crypt depth ratio
WBC: white blood cells count.
